# FKBP3, a poor prognostic indicator, promotes the progression of LUAD via regulating ferroptosis and immune infiltration

**DOI:** 10.1097/MD.0000000000038606

**Published:** 2024-06-28

**Authors:** Shengyi Li, Lexin Yang, Jing Li

**Affiliations:** a Internet of Things Engineering, Beijing-Dublin International College, Beijing University of Technology, Beijing, China; b State Key Laboratory of Protein and Plant Gene Research, College of Life Science, Peking University, Beijing, China.

**Keywords:** diagnosis, ferroptosis, FKBP3, immune infiltration, prognosis

## Abstract

**Background:**

Ferroptosis was reported to possess the therapeutic potentials in various human cancers. In the present study, we explored the expression, clinical significance and the molecular mechanism of FK506 binding protein 3 (FKBP3) in the progression of lung adenocarcinoma (LUAD).

**Material and Method:**

Cox regression was performed to obtain the prognosis related to differentially expressed genes (DEGs) in LUAD datasets from TCGA. We also downloaded the ferroptosis-related gene datasets from GeneCards. Venn diagram was performed to find the intersecting genes and FKBP3 was selected as the targeted gene by analyzing the diagnostic and prognostic values of Top10 intersecting genes. Moreover, univariate and multivariate analyses were performed to evaluate the association between clinicopathological factors and survival rates. GO/KEGG and GSEA analysis was performed to explore the function of FKBP3 in LUAD progression. Protein-protein interaction (PPI) network was performed via STRING database and the top10 hub genes were selected. Finally, the relationship between FKBP3 and immune infiltration was explored by ssGSEA analysis.

**Results:**

Firstly, 184 genes associated with the prognosis of LUAD and ferroptosis were obtained. FKBP3 was found to be significantly associated with a poor overall survival rate of LUAD patients. Immunohistochemical staining results showed that FKBP3 was highly located in cytoplasm and membrane of cells in LUAD tissues. PPI network analysis results showed that HDAC1, YY1, HDAC2, MTOR, PSMA3, PIN1, NCL, C14orf166, PIN4, and LARP6 were the top10 hub genes. Furthermore, spearman analysis results showed that the expression of FKBP3 was positively correlated with the abundance of Th2 cells and T helper cells.

**Conclusion:**

High level of FKBP3 was associated with poor prognostic outcomes of LUAD patients, which also inhibited immune infiltration in LUAD tissues. Additionally, FKBP3 was involved in regulating the ferroptosis process in LUAD patients. Thus, FKBP3 possessed the tumor promotion role might be involving in regulating ferroptosis and immune infiltration in LUAD progression.

## 1. Introduction

Lung cancer is a common and serious neoplastic disease that significantly threats human health in the world. Lung cancers can be divided into 2 types: small-cell lung cancer (SCLC) and non-small-cell lung cancer (NSCLC).^[1]^ Among them, NSCLC accounts for more than 85%, and lung adenocarcinoma (LUAD) is one of the most commonly subtype of lung cancers. At present, the clinical treatments of lung cancer include various methods such as surgical resection, chemotherapy, radiotherapy and molecular targeted therapy.^[2]^ The treatment of lung cancer mainly depends on the patient condition and individual differences.^[3]^ Therefore, the prevention and early detection of lung cancer is very important, which will help to improve the treatment effects and the survival rate of patients.

Ferroptosis, an iron-dependent lipid peroxidation regulated cell death, has been reported to be associated with various pathologies, such as ischemia-reperfusion injury, neurological diseases, and various human cancers.^[4–6]^ Ferroptosis normally possesses the tumor suppressor role in the progression of cancers. Induction of ferroptosis might be a promising approach to treat cancer, which could be a new way to overcome drug resistance in cancer therapy,^[7]^ as well as the key role in radiosensitization of lung cancers.^[8]^ Various genes or signaling pathways involves in promoting or inhibiting ferroptosis. For example, ferritin heavy chain 1 (FTH1), a marker of ferroptosis, was regulated by curcumenol via lncRNA H19/miR-19b-3p/FTH1 pathway.^[9]^ Butyrate promotes ferroptosis of lung cancer cells by regulating ATF3/SLC7A11 pathway.^[10]^ Isoorientin regulated ferroptosis and reversed cisplatin resistance by controlling the Nrf2/GPX4 pathway.^[11]^ Thus, it is crucial to clarify the key genes and molecular mechanisms that regulate ferroptosis in the progression of LUAD.

The TCGA database covers most cancers, including lung cancer, colorectal cancer, breast cancer, etc.^[12,13]^ At the same time, it also contains relatively comprehensive clinical information on tumor patients.^[14]^ Clinical information generally includes data such as patient survival time, survival status, gender, and clinical stage.^[15]^ In this study, we downloaded the gene set of the expression profile of LUAD tissue samples from the TCGA database, and screened the key genes related to the prognosis of LUAD, and downloaded it from the GeneCards database ferroptosis-related datasets. The intersecting genes with both prognosis of LUAD patients and ferroptosis were selected and the diagnostic, prognostic values, immune infiltration, as well as the possible molecular mechanism in LUAD progression were also investigated. It is very important to effectively identify the key pathogenic genes of LUAD for exploring its pathogenesis and studying the targeted therapy drugs.

## 2. Material and method

### 2.1. Data acquisition and processing

The RNAseq data and clinical data of LUAD patients were downloaded from TCGA database (https://portal.gdc.cancer.gov/). Till now, TCGA provided 539 LUAD samples and 59 normal lung tissue samples. All data were converted by log2 for subsequent analysis.

### 2.2. The top10 differentially expressed genes (DEGs) are analyzed in LUAD tissues and normal tissues

The top10 DEGs related to ferroptosis and prognosis of LUAD patients were analyzed by using R (4.2.1) software. Among them, the expression levels of Top10 genes were compared between LUAD tissues (n = 539) and normal tissues (n = 59) from TCGA database by Wilcoxon rank sum test.

### 2.3. Immunohistochemistry of FK506 binding protein 3 (FKBP3) in LUAD tissues and normal lung tissues

The cellular localization of FKBP3 was displayed by immunohistochemistry. Three representatives of specimens in LUAD tissues and normal lung tissues were shown to show the cell distribution of FKBP3 in the human protein atlas database (https://www.proteinatlas.org/).

### 2.4. Association of FKBP3 level and clinicopathological parameters

Correlation of FKBP3 expression and clinicopathological parameters of LUAD patients from TCGA database was performed according to the expression of FKBP3. Briefly, the total 539 LUAD patients were divided into high FKBP3 expression group and low FKBP3 expression group using the Wilcoxon rank sum test (continuous variables) or Pearson chi-square test (rank variables). The hazard ratio was also calculated by using univariate analysis and multivariate analysis.

### 2.5. Survival analysis in LUAD patients

The survival probability of high FANCI group and low FANCI group was analyzed in the Kaplan–Meier Plotter platform (www.kmplot.com), as well as in the Xiantao platform. The survival probability of subgroup analysis was also performed by gender, race, smoke, and TMN stages. The hazard ratio and its corresponding log-rank *P* value were provided for overall survival, disease-specific survival and progress-free interval.

### 2.6. GO/KEGG and GSEA analysis of FKBP3 in LUAD patients

Firstly, the correlations of FKBP3 level with all the other genes were analyzed with clusterProfiler [4.4.4] package of R (4.2.1)^[16,17]^ based on the whole transcriptome gene expression datasets in 539 LUAD patients from the TCGA project. The pathway enrichment analysis for GO/KEGG and GSEA analysis was performed in Xiantao platform(https://www.xiantaozi.com/). For GSEA analysis, we performed a difference analysis in correlation with FKBP3 in LUAD patients. Then, GSEA was carried out to elucidate the significant survival difference observed between high- and low-FKBP3 groups. Gene set permutations were performed 1000 times for each analysis. Signaling pathways were significantly enriched at false discovery rate (FDR) < 0.25 and p.adjust < 0.05.

### 2.7. A protein-protein interaction (PPI) network analysis and hub genes selection

A PPI network analysis of FKBP3 interacting genes was performed in STRING database (string-db.org). The medium confidence of 0.400 was selected as the minimum required interaction score. The max number of interactors showed no more than 20 interactors, and PPI enrichment *P* value was 6.5e-05. Cytoscape software was used to calculate the Top10 hub genes by using CytoHubba plug-in and ranked by MCC.

### 2.8. Relationship between FKBP3 and immune infiltration

Correlation between FKBP3 expression and immune infiltration in LUAD patients was assessed in a total of 24 immune cells. Briefly, single-sample GSEA (ssGSEA) approach and Wilcoxon rank sum test were performed to compare the difference between low FKBP3 group and high FKBP3 group in LUAD patients.

### 2.9. Statistical analysis

Statistical analysis was performed by using SPSS 21.0 (IBM Corp.). The differential expression of FKBP3 in LUAD and normal tissues was analyzed by Wilcoxon rank sum test. Univariate Cox proportional hazards regressions were applied to estimate the individual hazard ratio (HR) for the OS. Survival probabilities were performed by using the Kaplan–Meier method, and the differences between the survival curves were examined by the log-rank test. *P* < .05 was considered as a significant difference.

## 3. Results

### 3.1. Top10 genes related to ferroptosis and prognosis of LUAD patients are screened

**Figure 1.**
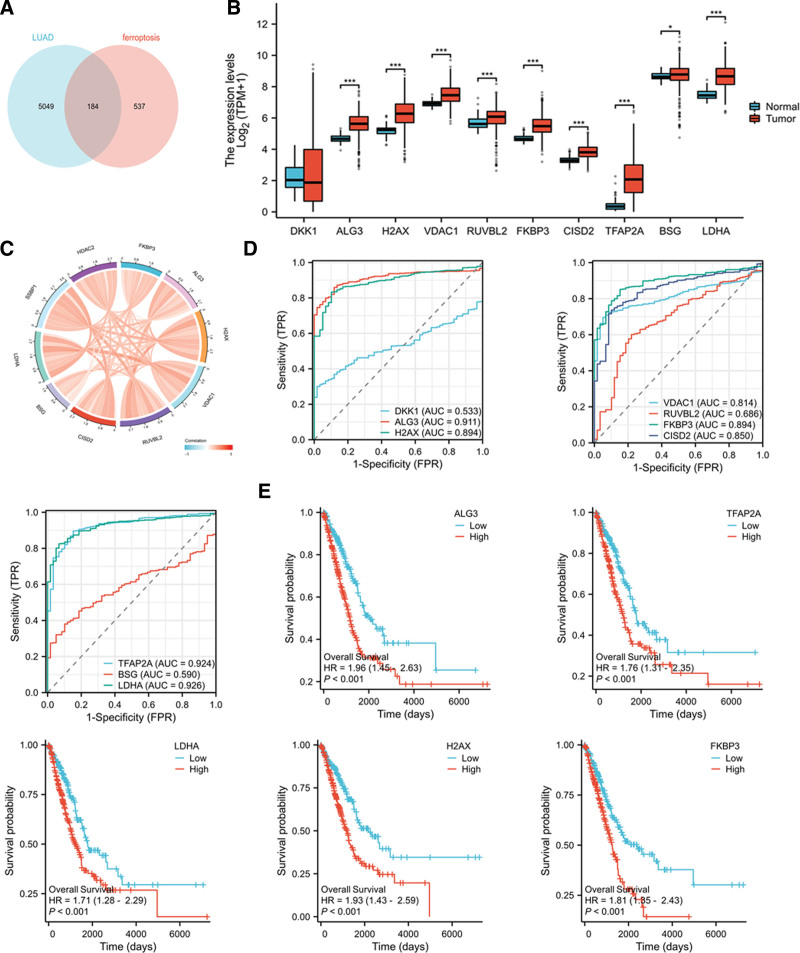
The ferroptosis-related DEGs for prognosis of patients with LUAD are screened. (A) Venn diagrams. There were 184 intersected genes in prognosis-related datasets for LUAD patients and ferroptosis-related datasets with gene score more than 1.00 from GeneCards. (B) The mRNA levels of top 10 genes correlated with the prognosis of LUAD and ferroptosis were shown in histogram. *P < .05, ***P < .001, compared with normal controls. (C) Chord diagram demonstrated a positive correlation between the top10 genes. (D) The diagnostic value of Top10 intersecting genes was investigated by ROC curve in normal lung tissue and LUAD. (E) Kaplan–Meier plotter was performed to detect the relationship between the expression of Top10 intersecting genes and the survival probability in LUAD patients. DEGs = differentially expressed genes, LUAD = lung adenocarcinoma.

It has been reported that modulating ferroptosis has a promising potential for cancer therapeutics.^[18]^ In order to explore whether ferroptosis was involved in the progression and prognosis of LUAD, the prognosis and ferroptosis-associated DEGs were first screened in LUAD patients. Briefly, RNAseq data were downloaded for the STAR process of the TCGA-LUAD project and the clinical data of LUAD patients were extracted in TPM format from TCGA database (https://portal.gdc.cancer.gov/). Overall survival analysis was performed by using survival packages [3.3.1] via batch-fitting survival Cox regression.^[19]^ We also downloaded the ferroptosis-related datasets from GeneCards (https://www.genecards.org/) and the ferroptosis-associated genes with gene scores more than 1.00 were selected. Venn diagram results showed that 184 genes associated with the prognosis of LUAD and ferroptosis (Fig. 1A). The expression of top10 intersecting genes was tested in TCGA-LUAD datasets and the results showed that the expression of ALG3, H2AX, VDAC1, RUVBL2, FKBP3, CISD2, TFAP2A, and LDHA were significantly upregulated in LUAD patients than that in normal controls (***<0.001, Fig. 1B). Moreover, we analyzed the correlation between 2 variables in the top 10 genes and visualized the correlation results by using the circle [v0.4.1] package. The spearman analysis results showed that there was a positive correlation between ferroptosis- and prognosis-associated top10 genes in LUAD patients from TCGA database (Fig. 1C, Table 1).

**Table 1:** Correlation coefficient table of Top 10 genes.

	FKBP3	ALG3	H2AX	VDAC1	RUVBL2	CISD2	BSG	LDHA	SSBP1	HDAC2
FKBP3		0.195	0.314	0.263	0.32	0.365	0.0756	0.367	0.364	0.383
ALG3	0.195		0.463	0.355	0.459	0.185	0.322	0.259	0.392	0.277
H2AX	0.314	0.463		0.349	0.525	0.334	0.277	0.358	0.435	0.417
VDAC1	0.263	0.355	0.349		0.345	0.42	0.267	0.543	0.382	0.405
RUVBL2	0.32	0.459	0.525	0.345		0.232	0.375	0.276	0.467	0.334
CISD2	0.365	0.185	0.334	0.42	0.232		0.131	0.492	0.469	0.353
BSG	0.0756	0.322	0.277	0.267	0.375	0.131		0.182	0.201	0.146
LDHA	0.367	0.259	0.358	0.543	0.276	0.492	0.182		0.509	0.381
SSBP1	0.364	0.392	0.435	0.382	0.467	0.469	0.201	0.509		0.386
HDAC2	0.383	0.277	0.417	0.405	0.334	0.353	0.146	0.381	0.386	

FKBP3 = FK506 binding protein 3.

### 3.2. FKBP3 is the interested gene with higher diagnostic and prognostic values in LUAD patients

Next, the diagnostic value of Top10 intersecting genes was investigated by ROC curve in normal lung tissue and LUAD. As shown in Figure 1D, the diagnostic values of Top10 intersecting genes further analyzed using a set of 539 LUAD patients and 59 normal controls. The results showed that the predictive ability of ALG3 (AUC = 0.911, CI = 0.887–0.936), TFAP2A (AUC = 0.924, CI = 0.895–0.953), and LDHA (AUC = 0.926, CI = 0.901–0.950) had high accuracy; The predictive ability of H2AX (AUC = 0.894, CI = 0.864–0.923), VDAC1 (AUC = 0.814, CI = 0.779–0.850), FKBP3 (AUC = 0.894, CI = 0.864–0.924) and CISD2 (AUC = 0.850, CI = 0.809–0.892) had moderate accuracy.

Next, Kaplan–Meier plotter was performed to detect the relationship between the expression of Top10 intersecting genes and the survival probability in LUAD patients. As shown in Figure 1E, high expression of ALG3, TFAP2A, LDHA, H2AX and FKBP3 were significantly associated with poor overall survival rate of LUAD patients. FKBP3 is a member of FK506-binding proteins (FKBPs). Till now, little is known on how FKBP3 regulating the progression of LUAD. Thus, we focused on the role and potential mechanism of FKBP3 in tumorigenesis and progression of LUAD.

### 3.3. Cellular localization of FKBP3 in LUAD tissues or normal control tissues

**Figure 2.**
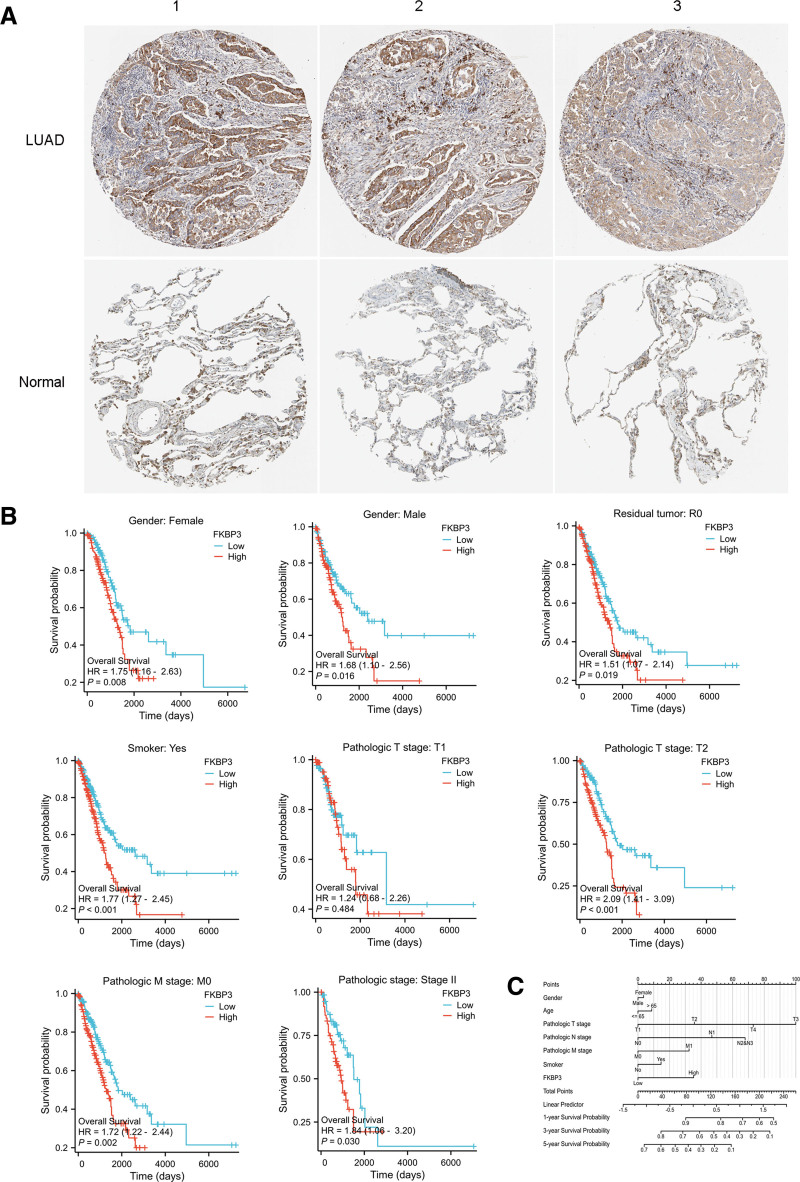
Subgroup analysis of FKBP3 and prognostic nomogram are performed in LUAD patients. (A) Immunohistochemistry of FKBP3 showed the FKBP3 distribution in LUAD tissues and the normal control lung tissues. Three representative images of FKBP3 expression were shown from human protein atlas database. (B) Subgroup analysis of prognostic potential of FKBP3 in LUAD patients. The survival probability was shown by Kaplan–Meier plotter analysis. (C) Nomogram for predicting probability of LUAD patients with 1-, 3-, and 5-yr overall survival. The 1-, 3-, and 5-yr related survival probabilities were obtained by draw a line straight down to the Risk axis. FKBP3 = FK506 binding protein 3, LUAD = lung adenocarcinoma.

We have detected that FKBP3 was significantly high expressed in LUAD tissues than normal controls and the high expression of FKBP3 was significantly associated with poor overall survival rate of LUAD patients. Next, we explored the cellular localization of FKBP3 in LUAD tissues and normal control tissues. The immunohistochemical staining results showed that FKBP3 was normally located in cytoplasmic and membranous position with high or medium staining in LUAD tissues, but in normal control the expression of FKBP3 was lowly expressed in cytoplasm and membrane of the lung tissues (Fig. 2A, Table 2).

**Table 2:** Immunohistochemistry of FKBP3 in human protein atlas database.

Tissue type	No.	ID	Age	Gender	Staining	Location
Normal	N1	2268	49	Female	Low	Cytoplasmic/membranous
	N2	2101	21	Male	Low	
	N3	2222	59	Male	Low	
LUAD	T1	2403	65	Female	High	Cytoplasmic/membranous
	T2	1847	64	Male	Medium	
	T3	1394	84	Male	Medium	

FKBP3 = FK506 binding protein 3, LUAD = lung adenocarcinoma.

### 3.4. Univariate and multivariate analysis of FKBP3 in LUAD patients

Next, the correlation between FKBP3 expression and clinicopathological parameters of LUAD was analyzed. Briefly, 539 cases were divided into 2 groups including low level of FKBP3 group (n = 269) and high expression of FKBP3 group (n = 270) and the baseline data are shown in Table 3. The mRNA level of FKBP3 was significantly correlated with OS event of LUAD patients (*P* = .004). However, there was no significant association between FKBP3 levels and other clinicopathological factors, such as gender (*P* = .894), age (*P* = .336), smoker (*P* = .780), pathologic stage (*P* = .101), pathologic T stage (*P* = .119), pathologic N stage (*P* = .059), pathologic M stage (*P* = .079). As shown in Table 4, the univariate analysis showed that a high level of FKBP3 was associated with the pathologic stage (*P* < .001), pathologic T stage (*P* < .001) and pathologic N stage (*P* < .001). Multivariate analysis results revealed that pathologic stage of stage IV (*P* = .031) and pathologic T stage of T3 (*P* = .004) were the independent factors with the overall survival time.

**Table 3:** Baseline data of LUAD patients in TCGA database.

Characteristics	Low expression of FKBP3	High expression of FKBP3	*P* value
n	269	270	
Gender, n (%)			894
Female	145 (26.9%)	144 (26.7%)	
Male	124 (23%)	126 (23.4%)	
Age, n (%)			.336
≤65	133 (25.6%)	124 (23.8%)	
> 65	125 (24%)	138 (26.5%)	
Smoker, n (%)			.780
No	37 (7%)	40 (7.6%)	
Yes	223 (42.5%)	225 (42.9%)	
Pathologic stage, n (%)			.101
Stage I	160 (30.1%)	136 (25.6%)	
Stage II	58 (10.9%)	67 (12.6%)	
Stage III	37 (7%)	47 (8.9%)	
Stage IV	9 (1.7%)	17 (3.2%)	
Pathologic T stage, n (%)			.119
T1	94 (17.5%)	82 (15.3%)	
T2	134 (25%)	158 (29.5%)	
T3&T4	39 (7.3%)	29 (5.4%)	
Pathologic N stage, n (%)			.059
N0	185 (35.4%)	165 (31.5%)	
N1	43 (8.2%)	54 (10.3%)	
N2&N3	30 (5.7%)	46 (8.8%)	
Pathologic M stage, n (%)			.079
M0	183 (46.9%)	182 (46.7%)	
M1	8 (2.1%)	17 (4.4%)	
OS event, n (%)			.004
Alive	189 (35.1%)	158 (29.3%)	
Dead	80 (14.8%)	112 (20.8%)	

FKBP3 = FK506 binding protein 3, LUAD = lung adenocarcinoma.

**Table 4:** The univariate and multivariate Cox regression analysis.

Characteristics	Total(N)	Univariate analysis		Multivariate analysis	
		Hazard ratio (95% CI)	*P* value	Hazard ratio (95% CI)	*P* value
Gender	530		.570		
Female	283	Reference			
Male	247	1.087 (0.816–1.448)	.569		
Race	472		.191		
Asian	8	Reference			
White	409	2.714 (0.380–19.403)	.320		
Black or African American	55	1.911 (0.254–14.382)	.529		
Age	520		.185		
≤65	257	Reference			
> 65	263	1.216 (0.910–1.625)	.186		
Pathologic stage	522		<.001		
Stage I	292	Reference		Reference	
Stage II	123	2.341 (1.638–3.346)	<.001	1.221 (0.646–2.309)	.539
Stage III	81	3.576 (2.459–5.200)	<.001	1.924 (0.724–5.118)	.190
Stage IV	26	3.819 (2.211–6.599)	<.001	2.435 (1.086–5.460)	.031
Histological type	483		.079		
Lung acinar adenocarcinoma	18	Reference		Reference	
Lung adenocarcinoma mixed subtype	109	3.194 (0.769–13.272)	.110	2.885 (0.687–12.116)	.148
Lung adenocarcinoma-not otherwise specified (NOS)	332	3.669 (0.908–14.832)	.068	3.622 (0.893–14.699)	.072
Lung bronchioloalveolar carcinoma mucinous	5	1.704 (0.154–18.802)	.664	3.337 (0.298–37.387)	.328
Lung bronchioloalveolar carcinoma nonmucinous	19	1.874 (0.363–9.678)	.454	2.677 (0.513–13.981)	.243
Pathologic T stage	527		<.001		
T1	176	Reference		Reference	
T2	285	1.507 (1.059–2.146)	.023	1.212 (0.836–1.757)	.310
T3	47	2.964 (1.762–4.986)	<.001	2.521 (1.347–4.720)	.004
T4	19	3.357 (1.767–6.376)	<.001	1.505 (0.696–3.256)	.299
Pathologic N stage	514		<.001		
N0	345	Reference		Reference	
N1	96	2.293 (1.632–3.221)	<.001	1.826 (1.001–3.329)	.050
N2&N3	73	2.993 (2.057–4.354)	<.001	1.430 (0.580–3.524)	.437
Smoker	516		.777		
No	74	Reference			
Yes	442	0.942 (0.625–1.420)	.775		
FKBP3	530		<.001		
Low	265	Reference		Reference	
High	265	1.812 (1.350–2.432)	<.001	1.732 (1.256–2.388)	<.001

FKBP3 = FK506 binding protein 3.

### 3.5. Subgroup analysis of FKBP3 and prognostic nomogram is performed in LUAD patients

The subgroup analysis was analyzed and shown in Figure 2B. In the female and male subgroup, the HR values were 1.75 (1.16–2.63) and 1.68 (1.10–2.56), and the corresponding *P* values were .008 and .016, respectively. In the residual tumor subgroup of R0 and smokers with LUAD, the HR values were 1.51 (1.07–2.14) and 1.77 (1.27–2.45), and corresponding *P* values of .019, and <.001, respectively. In the subgroup survival analysis performed with T2 stage, pathologic M stage of M0, and pathologic stage of Stage II cases, the HR values were 2.09 (1.41–3.09), 1.72 (1.22–2.44), and 1.84 (1.06–3.20), and the corresponding *P* values were <.001, .002, and .030 for the overall survival probability, respectively. In the other subgroups, there were no significant differences between the high and low FKBP3 groups.

Additionally, nomograms for predicting probability of LUAD with 1-, 3-, and 5-year overall survival were shown in Figure 2C. The prediction efficiency of the nomogram was also analyzed. The C-index of the OS nomogram was 0.687 (0.660–0.714); The likelihood ratio test was 53.62 on 10 df, *P* = 5.72e-08 suggesting the prediction efficiency of this model was moderately accurate.

### 3.6. GO and KEGG analysis of FKBP3 in LUAD patients

**Figure 3.**
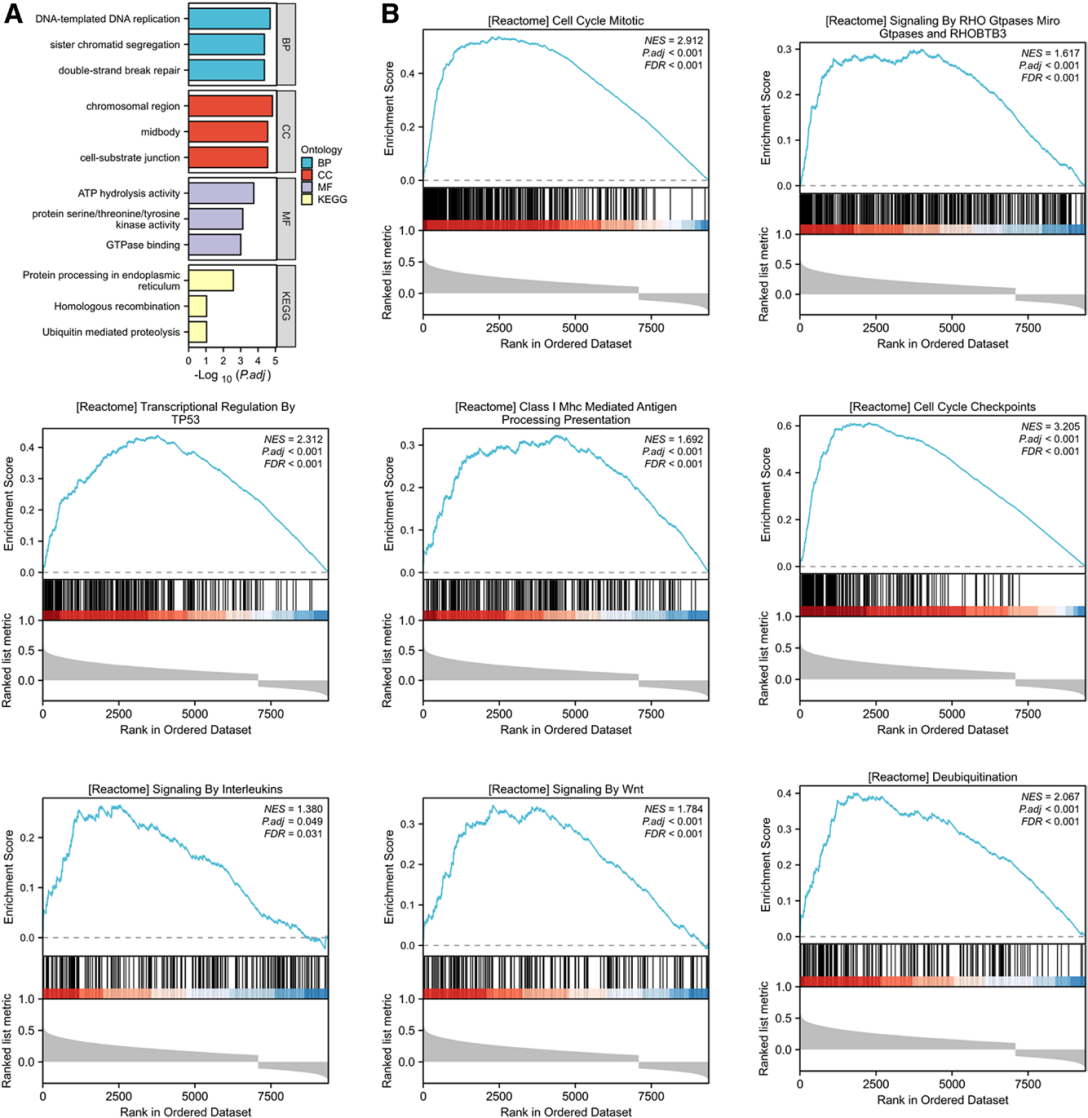
GO/KEGG and GSEA analysis of FKBP3 in LUAD patients. (A) GO and KEGG analysis of FKBP3 in LUAD progression. Biological process (BP); Molecular function (MF); Cellular component (CC); KEGG pathway analysis. (B) GSEA analysis of FKBP3. The results showed that FKBP3 was possibly involved in the signaling pathway including cell cycle mitotic, signaling by RHO GPTases miroGPTases and RHOBTB3, transcriptional regulation by TP53, class I Mhc mediated antigen processing presentation, cell cycle checkpoints, signaling by interleukins, signaling by Wnt and deubiquitination. FKBP3 = FK506 binding protein 3, LUAD = lung adenocarcinoma.

In order to explore the possible function of FKBP3 in the progression of LUAD, GO and KEGG analysis was performed by clusterProfiler [4.4.4] package. As shown in Figure 3A and Table 5, the results showed that FKBP3 co-expressed genes were mainly involved in DNA-templated DNA replication, sister chromatid segregation, and double-strand break repair etc in the biological process (BP) group. In the molecular function (MF) group, FKBP3 co-expressed genes were primarily enriched in ATP hydrolysis activity, protein serine/threonine/tyrosine kinase activity, GTPase binding etc. In the cellular component (CC) group, FKBP3 co-expressed genes were primarily enriched in chromosomal region, midbody, cell-substrate junction etc. KEGG pathway analysis demonstrated that FKBP3 co-expressed genes were enriched in protein processing in endoplasmic reticulum, homologous recombination and ubiquitin-mediated proteolysis.

**Table 5:** GO/KEGG analysis of FKBP3 in LUAD.

Ontology	ID	Description	GeneRatio	BgRatio	*P*	p.adjust
BP	GO:0006261	DNA-templated DNA replication	28/925	159/18800	3.78e-09	1.99e-05
BP	GO:0006302	Double-strand break repair	37/925	273/18800	2.24e-08	4.26e-05
BP	GO:0000819	Sister chromatid segregation	31/925	205/18800	2.42e-08	4.26e-05
BP	GO:0007059	Chromosome segregation	42/925	348/18800	7.56e-08	8.15e-05
BP	GO:0048285	Organelle fission	53/925	493/18800	7.74e-08	8.15e-05
CC	GO:0098687	Chromosomal region	44/943	366/19594	2.23e-08	1.47e-05
CC	GO:0030055	Cell-substrate junction	47/943	428/19594	1.21e-07	2.75e-05
CC	GO:0030496	Midbody	29/943	203/19594	1.51e-07	2.75e-05
CC	GO:0005925	Focal adhesion	46/943	419/19594	1.67e-07	2.75e-05
CC	GO:0000779	Condensed chromosome, centromeric region	24/943	156/19594	4.47e-07	5.91e-05
MF	GO:0016887	ATP hydrolysis activity	40/932	325/18410	1.91e-07	0.0002
MF	GO:0004712	Protein serine/threonine/tyrosine kinase activity	47/932	446/18410	1.65e-06	0.0008
MF	GO:0051020	GTPase binding	35/932	298/18410	3.25e-06	0.0010
MF	GO:0044389	Ubiquitin-like protein ligase binding	36/932	317/18410	5.12e-06	0.0011
MF	GO:0048156	Tau protein binding	11/932	43/18410	6.82e-06	0.0011
KEGG	hsa04141	Protein processing in endoplasmic reticulum	27/516	171/8164	8.17e-06	0.0027
KEGG	hsa04120	Ubiquitin-mediated proteolysis	20/516	142/8164	.0006	0.0921
KEGG	hsa03440	Homologous recombination	9/516	41/8164	.0009	0.0921

FKBP3 = FK506 binding protein 3, LUAD = lung adenocarcinoma.

### 3.7. GSEA analysis demonstrates FKBP3-related signaling pathways in LUAD patients

Next, we investigated the possible signaling pathways that FKBP3 regulated in LUAD progression, GSEA analysis was performed and the results showed that there were 607 signaling pathways significantly enriched at false discovery rate (FDR) < 0.25, p.adjust < 0.05. The differentially enriched terms were including cell cycle mitotic, signaling by Rho GTPases miroGTPases and RhoBTB3, transcriptional regulation by TP53, class I MHC mediated antigen processing presentation, cell cycle checkpoints, signaling by interleukins, signaling by Wnt, and deubiquitination etc (Fig. 3B).

### 3.8. PPI network analysis of FKBP3

**Figure 4.**
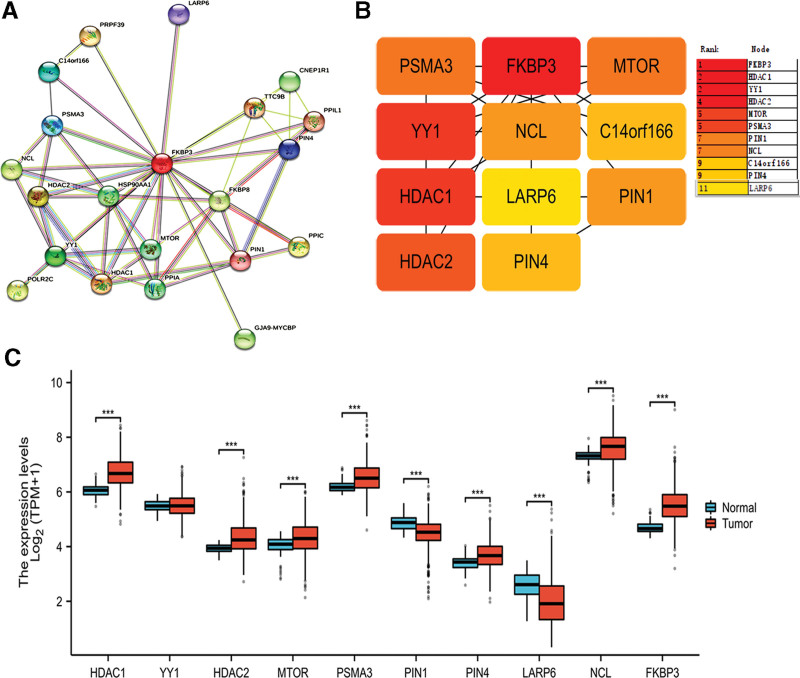
The analysis of top 10 hub genes correlated with FKBP3. (A) PPI analysis of the interacting proteins with FKBP3 in homo sapiens via STRING database. (B) Cytoscape software was used to analyze the PPI network. The top 10 hub genes were clarified by using CytoHubba plug-in and ranked by MCC. (C) The relative expression of hub genes in LUAD tissues and normal control tissues was shown. Wilcoxon rank sum test was performed. ***P < .001. FKBP3 = FK506 binding protein 3, LUAD = lung adenocarcinoma, PPI = protein-protein interaction.

Next, we analyzed the interacting proteins of FKBP3 in homo sapiens, the PPI network was constructed in STRING database. Here, the medium confidence of 0.400 was selected as the minimum required interaction score. The maximum number of interactors showed no more than 20 interactors. The number of nodes was 21, the number of edges was 55, average node degree was 5.24, avg.local clustering coefficient was 0.703, expected number of edges was 31 and PPI enrichment *P* value was 6.5e-05 (Fig. 4A). Moreover, top 10 hub genes were predicted by using Cytoscape, which included HDAC1, YY1, HDAC2, MTOR, PSMA3, PIN1, NCL, C14orf166, PIN4, and LARP6 (Fig. 4B). Moreover, the expression of hub genes was detected in LUAD tissues and normal controls in TCGA database. As shown in Figure 4C, the expression of HDAC1, HDAC2, MTOR, PSMA3, PIN4, NCL and FKBP3 were significantly upregulated in LUAD tissues than that of control tissues (Wilcoxon rank sum test, ***P < .001). However, the expression of PIN1 and LARP6 was significantly decreased in LUAD tissues than that of control tissues (Wilcoxon rank sum test, ***P < .001). Thus, FKBP3 might regulate the progression of LUAD by interacting with the hub genes.

### 3.9. Relationship between FKBP3 and immune infiltration

**Figure 5.**
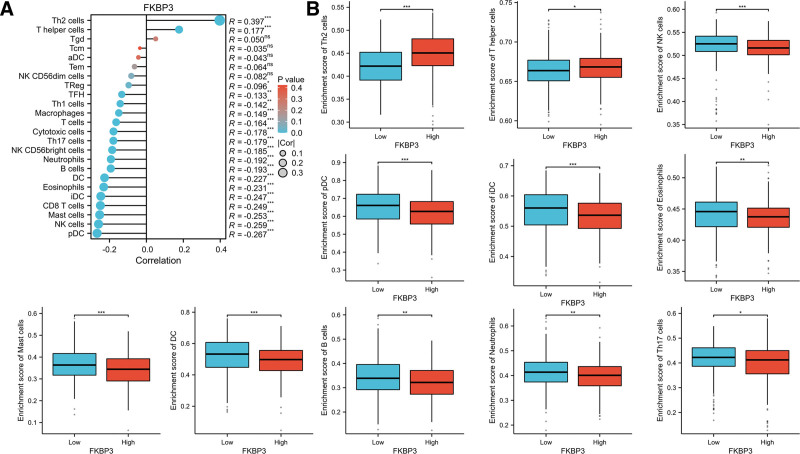
Relationship between FKBP3 and immune infiltration. (A) Immune infiltration lollipop chart of FKBP3 in LUAD patients. (B) The correlation between the expression level (TPM) of FKBP3 and enrichment scores of immune cells quantified by ssGSEA analysis. *P < .05, **P < .01, ***P < .001, compared with the group with low expression of FKBP3. FKBP3 = FK506 binding protein 3, LUAD = lung adenocarcinoma.

Furthermore, to explore the correlation between FKBP3 expression and immune infiltration, we investigated the association between FKBP3 expression and immune infiltration was tested by using spearman analysis and visualized with ggplot2 packages.^[20]^ As shown in Figure 5A, spearman analysis results showed that the expression of FKBP3 was positively correlated with the abundance of Th2 cells (R = 0.398, *P* < .001) and T helper cells (R = 0.131, *P* < .01), and negatively correlated with the abundance of pDC (R = −0.220, *P* < .001), mast cells (r = −0.211, *P* < .001), NK cells (r = −0.189, *P* < .001), iDC (r = −0.185, *P* < .001), eosinophils (r = −0.184, *P* < .001), CD8 T cells (r = −0.173, *P* < .001), DC (r = −0.171, *P* < .001), B cells (r = −0.145, *P* < .001), and Th17 cells (r = −0.133, *P* < .001). According to the median value of FKBP expression in LUAD patients from TCGA database, the cases were divided into 2 groups: low level of FKBP3 group (n = 269) and high expression of FKBP3 group (n = 270). The immune infiltration results analyzed by Wilcoxon rank sum test demonstrated that high expression of FKBP3 had significantly higher enrichment scores of Th2 cells (***P < .001) and T helper cells (*P < .05); However, the enrichment scores of NK cells, pDC, iDC, mast cells and DC cells were significantly decreased in high expressed FKBP3 group than that of the low expressed FKBP3 group (***P < .001, Fig. 5B). All the data revealed that higher expression of FKBP3 inhibited the immune infiltration of antitumor related immune cells.

## 4. Discussion

LUAD was a major subtype of NSCLC, which was the leading cause of mortality of lung cancers.^[21]^ Although ferroptosis was considered as the effective potential to overcome cancer progression, till now, the exact mechanism of ferroptosis in LUAD progression remains elusive.^[22,23]^ In the present study, we downloaded the RNAseq data and clinical data of human LUAD from TCGA and Wilcoxon rank sum test was performed to selected the prognosis-related DEGs in LUAD patients. We also downloaded the ferroptosis-related datasets from GeneCards and the intersecting genes were selected, including ALG3, H2AX, VDAC1, RUVBL2, FKBP3, CISD2, TFAP2A and LDHA, all of which were involved in the ferroptosis of cancer cells. It has been reported FKBP3 regulated oxalipalatin resistance via regulating HDAC2 expression in colorectal cancers.^[24]^ It also promoted the NSCLC progression via Sp1/HDAC2/p27.^[25]^ However, the exact role and molecular mechanism of FKBP3 were not clearly clarified in LUAD progression. In the present study, spearman analysis was performed and the results showed that FKBP3 had a positive correlation with H2AX (*R* = 0.314, *P* = 3.03e-13), VDAC1(*R* = 0.263, *P* = 1.21e-09), RUVBL2 (*R* = 0.32, *P* = 1.01e-13), CISD2 (*R* = 0.365, *P* = 0), LDHA (*R* = 0.367, *P* = 0), SSBP1 (*R* = 0.364, *P* = 0). It has been reported that inhibiting ALG3 increased the lipid accumulation and immunogenic ferroptosis of cancer cells.^[26]^ In gastric cancer, B cell receptor associated protein 31 (BAP31) knockdown increased cellular ferroptosis by binding to VDAC1 and affecting its polyubiquitination.^[27]^ Moreover, inhibiting VDAC1 oligomerization inhibited ferroptosis in Acetaminophen (APAP)-induced liver injury.^[28]^ RUVBL1/2 was highly overexpressed in NSCLC and its ATPase activity was necessary in DNA replication during cancer progression.^[29]^ FKBP3 was a member of FK506-binding proteins (FKBPs) family and correlated with the activity of histone deacetylase 2 (HDAC2) through regulation PTEN/AKT pathway.^[24]^ It also promoted proliferation of NSCLC cells via Sp1/HDAC1/p27 pathway.^[25]^ LDHA was also involved in ferroptosis and glycolysis in prostate cancer via Nrf2 pathway,^[30]^ and regulating the immunity response in colorectal cancer.^[31]^ Thus, we speculated that FKBP3 might regulated the proliferation of LUAD cells via regulating ferroptosis, which need to be further validated by in vitro experiments in near future.

Firstly, we explored the clinical significance of FKBP3 in LUAD patients from TCGA database. Wilcoxon rank sum test analysis was performed between 539 specimens in LUAD group and 59 samples in normal control group, the results revealed that the expression of FKBP3 mRNA was significantly higher in LUAD tissues than in the normal controls. Moreover, the cellular distribution of FKBP3 was validated by immunohistochemical staining from HPA database. The results showed that FKBP3 was highly expressed in LUAD tissues and lowly expressed in normal control lung tissues. It was normally located in cytoplasmic and membranous positions with high staining in LUAD tissues. Additionally, univariate analysis was performed that a high level of FKBP3 was closely associated with the pathologic stage, pathologic T stage and pathologic N stage. The diagnostic value of FKBP3 was also validated and the results showed FKBP3 had moderate prediction accuracy and effectiveness in diagnosing LUAD. All these results demonstrated that FKBP3 had an important clinical significance in the prognostic and diagnostic value in LUAD patients.

Next, we explored the possibly molecular mechanisms that FKBP3 might involve in the progression of LUAD. GO/KEGG and GSEA analysis results showed that FKBP3 was probably involved in protein processing in endoplasmic reticulum, cell cycle mitotic and cell cycle checkpoints etc. We also found that FKBP3 was involved in DNA-templated DNA replication. It was consistent with the results from Dilworth, who thought that FKBP3 participated in DNA double-strand break repairing.^[32]^ The GSEA analysis also demonstrated that FKBP3 was involved in transcriptional regulation by TP53, which was also proved and confirmed by the reports that FKBP3 induced the degradation of MDM2 and activation of p53 as the novel regulator of the p53 signaling pathway.^[33]^ Moreover, FKBP3 promoted the proliferation of NSCLC cells by interacting with HDAC2, which modulated the acetylation of histone H3K4 and inhibited by p27.^[25]^ Importantly, the exact mechanisms of FKBP3 in regulating the ferroptosis of LUAD cells need to be further explored.

Furthermore, the correlation between FKBP3 and immune infiltration was also explored by using spearman analysis in LUAD datasets from TCGA. The results showed that FKBP3 was positively correlated with the abundance of Th2 cells. As we known, Th2 response was crucial for pro-tumorigenic effects and promoting tumor progression,^[34]^ suggesting that FKBP3 might promote the tumor escape in the progression of cancers. Simultaneously, the abundance of NK cells, CD8 T cells, DC cells were significantly inhibited in high expression of FKBP3 group of LUAD patients. Th1 type response contributed to antitumor response in cancer development and associated with improved overall survival of cancer patients.^[35]^ All these results demonstrated that overexpression of FKBP3 promoted LUAD progression partly by inhibiting the Th1 type response and promoting Th2-type immune response.

In conclusion, we found that in LUAD patients, overexpression of FKBP3 was closely related to poor prognostic outcome and FKBP3 had a moderate diagnostic value in LUAD patients. Mechanically, FKBP3 promoted the tumor progression by regulating the ferroptosis and inhibited immune infiltration in LUAD progression.

## Author contributions

**Conceptualization:** Jing Li.

**Data curation:** Shengyi Li.

**Formal analysis:** Lexin Yang.

**Investigation:** Shengyi Li, Lexin Yang, Jing Li.

**Methodology:** Jing Li.

**Project administration:** Shengyi Li, Lexin Yang.

**Supervision:** Jing Li.

**Validation:** Shengyi Li, Lexin Yang.

**Visualization:** Lexin Yang.

**Writing – original draft:** Jing Li.

**Writing – review & editing:** Jing Li.
